# Factors that Influence Work Family Conflict for Women Faculty

**DOI:** 10.15694/mep.2021.000063.1

**Published:** 2021-03-08

**Authors:** Julie Radico, Tamara K. Oser, Tracy B. Fausnight, Arthur Berg, Ann Ouyang, Shou Ling Leong

**Affiliations:** 1 Penn State College of Medicine and Penn State Health; 2 University of Colorado School of Medicine Anschutz and the Penn State College of Medicine; 3 Penn State College of Medicine

**Keywords:** Women Faculty, Departmental Culture, Primary Care, Work Family Conflict, Academic Medicine

## Abstract

This article was migrated. The article was marked as recommended.

**Purpose:** This study examined the interaction between work role overload, work-to-family conflict, and departmental/division culture conducive to women’s academic success.

**Methods:** All women assistant and associate professors eligible for promotion from the Departments of Family Medicine, Internal Medicine, and Pediatrics were invited to complete a validated web-based survey that measured work-to-family conflict, work hours, work role overload, and culture conducive to women’s academic success ( Westring *et al.,* 2012).

**Results:** With 88 survey respondents, high work role overload was associated with increased levels of work-to-family conflict while those who reported a higher culture conducive to women’s academic success reported less work-to-family conflict. Culture conducive to women’s academic success did not moderate the impact of work demand on work-to-family conflict.

**Conclusions:** While departmental/division culture was important, it was not sufficient to completely mitigate work-to-family conflict. Work demand appears to impact work-to-family conflict related to strain, in which women report being too stressed by work to focus on their family and their own health and wellness. Employers can greatly impact work culture by reducing the strain of work demands that interfere with women pursuing promotion, increase burnout, and contribute to women faculty deciding to work part-time.

## Introduction

While the numbers of women graduating from medical school and working in academic medicine are increasing, women are promoted to associate and full professor at significantly lower rates than their male counterparts as noted as recently as 2019 ( Richter *et al.,* 2020; Nonnemaker, 2000; Jena *et al.,* 2016; Lautenberger *et al.,* 2014; Blumenthal *et al.,* 2017; Bennett *et al.,* 2019). The gender discrepancy remains evident even when accounting for age, experience, graduation year, race or ethnic group, and specialty ( Richter *et al.,* 2020; Jena *et al.,* 2015; Carr *et al.,* 2018). Women tend to hold fewer senior academic appointments (e.g. administration, chair, and dean positions) ( Lautenberger *et al.,* 2014; Tulunay-Ugur *et al.,* 2019; Schor, 2018; Jena *et al.,* 2016) and, overall, women earn nearly $20,000 less than their male counterparts annually even after accounting for age, experience, specialty, faculty rank, measures of research productivity, and clinical revenue ( Jena *et al.,* 2016). Adding further to this complexity, women in academic medicine often have greater responsibilities outside of their careers, spending an average 8.5 more hours per week on domestic activities and, furthermore, perceive their work environment as less supportive in helping them manage the demands of work and responsibilities outside the workplace ( Carr *et al.,* 1998; Reed and Buddeberg-Fischer, 2001; Pololi *et al.,* 2013; Jolly *et al.,* 2014).

Compared to men, full-time women in academic medicine tend to perceive lower gender equity, are less likely to believe their institutions are making changes to address diversity goals or view their institution as family-friendly ( Pololi *et al.,* 2013). Physician mothers commonly experience discrimination including, disrespectful treatment by nursing or other support staff, not being included in administrative decision making, and inequity with regard to pay and benefits compared with their male peers ( Adesoye *et al.,* 2017). Paid family leave policies are often lacking even at “top-tier medical schools” which fall short of the 12 weeks recommended by The American Academy of Pediatrics averaging only 8.6 weeks ( American Academy of Pediatrics, 2017; Riano *et al.,* 2018). Moreover, parenting was reported to adversely affect promotion among women but not men and female physicians with children have increased odds of refusing a project or committee participation ( Morgan *et al.,* 2020).

In 2010, research was conducted with baseline data collected among early career women faculty whichdemonstrated a critical role of the department and division culture on women faculty careers ( Westring *et al.,* 2014). The faculty were from 27 departments/divisions with 3 or more assistant professors at a single, urban, research institution. A supportive departmental/division work culture ( Westring *et al.,* 2012) has been shown to have a mitigating effect on the negative impact of work role overload ( Peterson *et al.,*1995; Westring *et al.,* 2014) and long work hours on levels of work-to-family conflict ( Carlson *et al.,* 2000; Westring *et al.,* 2014). The purpose of this study is to investigate the work-to-family conflict at our suburban academic medical center. We aim to identify relationships between the work demands (WD); WD include: work role overload and work hours, work-to-family conflict, and departmental culture for women in three departments: Family Medicine (FM), Internal Medicine (IM) and Pediatrics (PEDS). We hope to identify ways in which a positive departmental culture reduces work-to-family conflict.

## Methods

This study was approved by the Penn State College of MedicineInstitutional Review Board, STUDY00007918, 11/17/2017.

### Survey of women faculty who are eligible for promotion

From November 2017-February 2018, we invited all women assistant and associate professors from the Penn State College of Medicine Departments of FM, IM, and PEDS to complete a validated web-based survey delivered through REDCap that measured work hours, work-to-family conflict, department/division culture, and work role overload. These departments were chosen as they are all providers of primary care.

The survey included validated measures of work-to-family conflict ( Carlson *et al.,* 2000) and culture ( Westring *et al.,* 2012), as well as socio-demographic factors (hometown, age), academic work history (department and appointment), marital status (single, married/domestic partnership, divorced), children (do you have children, yes/no), and work hours (how many hours per week, on average, do you spend in work-related activities?) ( Grisso *et al.,* 2017). Since the number of racial/ethnic minority faculty is so low, the two survey questions related to race and ethnicity were written such that these particular respondents would not be self-identifying. The questions, therefore, asked if respondents felt race and ethnicity are barriers for advancement of women faculty and, if they had personallyexperienced or witnessed race and ethnicity acting as barriers for advancement of women faculty. ( Table 1).

**Table 1:** Characteristics of 88 Women Assistant and Associate Professors, 2018

Characteristic	Measure
**Age in years (n=88)**	**no. (%)**
30 and under	2 (2.27%)
31-40	33 (37.5%)
41-50	29 (33%)
51-60	21 (23.9%)
Over 61	3 (3.4%)
**Do you have children? (n=88)**	**no. (%)**
Yes	69 (78.4%)
No	19 (21.6%)
**Marital status (n=87)**	**no. (%)**
Single	7 (8.1%)
Married or domestic partnership	76 (87.4%)
Divorced	4 (4.6%)
**Race (n=88)**	**no. (%)**
Do you feel race and ethnicity are barriers for advancement of women faculty?	34 (39%)(Yes responses)
Have you personally experienced or witnessed race and ethnicity acting as barriers for advancement of women faculty?	14 (16%)(Yes responses)
**Appointment (n=87)**	**no. (%)**
Full-time	72 (82.8%)
Part-time	15 (17.2%)
**Hometown (n=88)**	**no. (%)**
Suburban	53 (60.2%)
City	12 (13.6%)
Rural	23 (26.1%)
**Does your spouse work outside of the home? (n=76)**	**no. (%)**
Yes	66 (86.8%)
No	10 (13.2%)
**Number of hours your partner works outside the home (n=65)**	**no. (SD)**
	52.1 (14.7)

### Work-to-family conflict

Two subscales from a multidimensional measure of conflict between work and family ( Carlson *et al.,* 2000), described by Westring *et al.* (2014), were included. Time-based work-to-family conflict is defined as when the time demands of work (e.g., long hours) interfere with effective participation in the family role, whereas strain-based work-to-family conflict is when the stress or strain from work has a negative effect on family life ( Westring *et al.,* 2014). The subscales consisted of three items and were scored on a five-point scale from 1 (strongly disagree) to 5 (strongly agree) ( Westring *et al.,* 2014).

### Culture conducive to women’s academic success

Culture is defined as the shared perceptions/beliefs of the respondents regarding the extent to which the department or division culture is supportive of women’s careers ( Westring *et al.,* 2012). The measure consists of 45 items covering four dimensions of culture for women’s careers: support for work-life balance, equal access to opportunities, freedom from gender bias, and chair/chief support ( Westring *et al.,* 2014). All items are rated on a 5-point scale from 1 (strongly disagree) to 5 (strongly agree). For IM and PEDS the word “division” replaced “department” and “chief” replaced “chair” throughout the survey ( Westring *et al.,* 2012) as the authors from these departments identified that division chiefs had more of a direct impact on the culture experienced by women faculty.

### Work role overload

We used the three work role overload questions from Peterson *et al.*’s (1995) role overload scale previously selected by Westring *et al.* (2014). The items are: “I feel overburdened by my work responsibilities, the amount of work I have to do interferes with the quality of work I want to maintain, and my workload is too heavy.” Each item was rated on a five point scale from 1 (strongly disagree) to 5 (strongly agree).

### Statistical analysis

All statistical analyses and graphics were produced in a reproducible report using the R statistics program (version 3.6.0) with Knitr package. Univariate tables were constructed with the CompareGroups package. Other statistical methods are described in the respective sections below.

## Results/Analysis

The sample consisted of 88 respondents, out of 183 (response rate=48%) eligible women, with 25 out of 31 eligible participants from FM (80.6%), 26 out of 80 from IM (32.5%), and 37 out of 72 from PEDS (51.4%).

The characteristics of the participants are described in Table 1. Careful consideration was given as to how questions were asked so as to minimize the possibility that individual participants could be identified (i.e. “do you have children?” was asked rather than “how many children do you have?”).

### Correlations based on survey results

Work role overload and work-to-family conflict were positively correlated, the higher the work role overload, the higher the perceived work-to-family conflict (r=0.71; p=<0.001). Conversely, participants who reported low work role overload also reported low work-to-family conflict. There was a correlation with hours worked and work-to-family conflict (p=0.004).

Culture and work role overload were negatively correlated, the higher the culture was perceived as conducive to women’s academic success the lower the work role overload (r=-0.36, p=<0.001).

Work-to-family conflict was found to be similarly negatively correlated with culture; the higher the culture conducive to women’s academic success the lower the work-to-family conflict (r=-0.48; p=<0.001). Conversely, on average, women who reported a less supportive work culture reported high work-to-family conflict.

### Culture conducive to women’s academic success

The relationship of the four dimensions of culture to work-to-family conflict was evaluated by comparing the scores of items in these dimensions in the cohort of women faculty that had work-to-family conflict scores above the median and below the median. A low scale for work-to-family conflict was 0-3.71 and a high scale was 3.72 to 5, with 44 faculty in each group. The following identifies significant items in each of these four areas of the culture related to low or high work-to-family conflict. The statistical analysis was conducted using a t-test for continuous variables and a chi squared test for categorical variables.

### Equal access to opportunities

Those with high work-to-family conflict reported that they did not receive adequate mentoring from senior faculty (p=0.001), feedback regarding their performance (p=0.002), or guidance about potential research opportunities (p=0.003). They also reported not having protected time for research (p=0.001), not being properly recognized for their work (p=<0.001), not being offered opportunities to sit on prestigious committees (p=0.002), nor being frequently nominated for awards and honors (p=0.003).

### Support for work-life balance

Those with high work-to-family conflict reported that they do not feel family demands are considered when the department develops schedules for teaching and clinical hours (p=0.001) and also feel that work is expected to be the primary focus of faculty members’ lives (p=0.001).

### Freedom from gender bias

Those with high work-to-family conflict reported feeling uncomfortable raising issues about the supportiveness of the work environment (p=0.003).

### Chair/chief support

Respondents with lower work-to-family conflict agreed that their chair/chief tries to ensure that faculty are able to manage the demands of work and family (p=0.003) and that their chair/chief tries to ensure that faculty feel free to express concerns regarding their treatment (p=0.009).

Those with lower perceived chair/chief support reported increased strain-based work-interference with their family (p=0.04) (e.g. “stress from work makes it harder for me to be fully involved in my family”) and increased strain-based work-interference with their health (p=0.003) (e.g. “due to all the pressures from work, sometimes I am too stressed to engage in health and wellness-related activities”). Overall, when the chair/chief support was perceived as higher, the higher the culture (p=<0.001).

### Moderation analysis

We evaluated the moderating effect of department on the relation between work role overload and work-to-family conflict. Culture and work role overload were mean centered to reduce statistical issues related to being highly correlated. A Box-Cox power transformation was applied to work-to-family conflict to stabilize the variance and reduce skewness (i.e. to make the work-to-family conflict distribution closer to normal distribution). Culture was not found to have a statistically significant moderation effect with regard to work role overload on work-to-family conflict (p=0.47); no statistically significant interaction effect was found between work role overload and culture on work-to-family conflict. Though work role overload and culture were statistically significantly associated with work-to-family conflict (p=<0.001; 0.003). Also, there were no significant findings when examined by department (FM, IM, PEDS).

**Figure 1:**
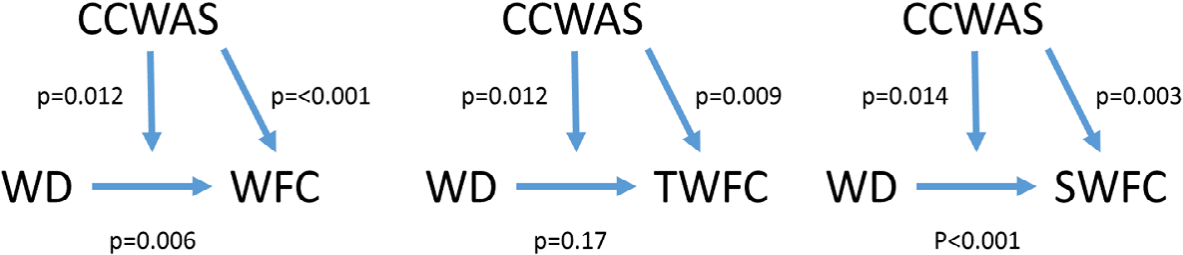
Moderation Analysis of culture, work role overload, work-to-family conflict (Overall), Twork-to-family conflict (Time-based), Swork-to-family conflict (Strain-based)

In a multivariate analysis that includes WD (i.e. work role overload and work hours combined) and culture on work-to-family conflict, both WD and culture were marginally statistically significant. However, the interactions (moderation effect) were not statistically significant (p=0.12). Similar results hold when work hours are removed and the analysis solely includes work role overload. Although not statistically significant at the sample size explored in this study, the results show some moderating effect of culture on the relationship between WD and work-to-family conflict. It is interesting to note, that when we analyze strain-based work-to-family conflict and time-based work-to-family conflict separately, we find that WD was only statistically significantly associated with strain-based work-to-family conflict (p<0.001) but not time-based work-to-family conflict (p=0.17). Culture, however, was significantly associated with both time-based work-to-family conflict (p=0.009) and strain-based work-to-family conflict (p=0.003). These results are collectively depicted in **Figure 1.** Strain-based work-to-family conflict includes feeling: “frazzled,” “stressed,” and “pressure” which may be key components to contributing to work-to-family conflict. We do not see this with time-based work-to-family conflict, which could mean that strenuous components of work (e.g. stress) might be more strongly perceived to have an impact of work-to-family conflict, than a high amount of work hours.

The p-values depicted in the graphs and derived from a multivariate linear regression model of culture and work role overload on work-to-family conflict, Twork-to-family conflict, and Swork-to-family conflict including an interaction effect.

**Abbreviations:** CCWAS: Culture Conducive to Women’s Academic Success, WD: Work Demand (Work Demand is a combination of work role overload and work-to-family conflict), WFC: Work-to-Family Conflict, TWFC: Time-based Work-to-Family Conflict, SWFC: Strain-based Work-to-Family Conflict.

### Part-time employment

Having children did not affect perceived work-to-family conflict (p=0.60). However, having children was significantly associated with faculty appointment status (part or full-time) (p=0.03). All participants (100%) who were part-time (n=15) had children, whereas 73.6% of full-time participants had children (n=53). Of the faculty who responded to the survey and self-identified as part-time (n=15) seven were from PEDS, one was from IM, and seven were from FM. Of note all faculty who self-identified as single (FM=2, IM=4, PEDS=1, n=7) or divorced (FM=2, PEDS=2, n=4) were full-time.

Considering work-to-family conflict on a scale of low (0-3.33), medium (3.33-4), and high (4-5), with an approximately equal number of faculty in each group, we identified that employment status (full/part-time) had borderline statistical significance. Part-time participants reported less work-to-family conflict (p=0.06). Those who worked part-time reported being more satisfied with their family life (p=0.02) and having better overall mental (p=<0.001) and physical health (p= 0.001) in the last 6 months. Part-time workers also reported working an average of 41.9 (SD=9.97) hours a week compared to full-time faculty 59.2 (SD=10.6) hours (p=<0.001). In addition, part-time workers reported overall lower stress levels (p= 0.01) in the last 6 months and higher overall life satisfaction (p=<0.011).

## Discussion

In our efforts to identify relationships between the work demands (work role overload and work hours), work-to-family conflict, and departmental culture conducive to women’s academic success, we discovered mixed results.

We found that high work role overload was associated with increased levels of work-to-family conflict and those who reported lower work-to-family conflict reported higher departmental/division culture. Those with high work-to-family conflict reported that they did not have equal access to opportunities, support for work-life balance, or freedom from gender bias (e.g. felt uncomfortable raising issues about the supportiveness of the work environment). Conversely, respondents with low work-to-family conflict agreed that their chair/chief tries to ensure that faculty are able to manage the demands of work and family (p=0.003).

While we found correlations between culture, work-to-family conflict, and work role overload, the departmental/division culture did not significantly moderate (reduce) the impact of WD (work role overload and work hours) on work-to-family conflict. In a moderation analysis we found that WD was associated with strain-based work-to-family conflict. It appears that high work role overload and longer work hours (i.e. WD) are associated with women feeling too frazzled and stressed to fully engage with and enjoy time with their family or focus on their own health and wellness. The emotional drain from work has an impact on how much mental and emotional reserve women have to spend on their family or themselves. This is especially notable as strain-based work-to-family conflict may be compounded by how women are still often tasked with being the main caretaker for the family and the home, while feeling too stressed from work to fully engage with their family and/or in health focused activities. Women may be faced with the decision to prioritize family at the expense of career milestones, including promotion and leadership positions, especially when institutional policies or practices conflict with their values concerning work-life balance ( Jones *et al.,* 2016). Women more frequently report caregiving responsibility for other family members (e.g. aging parents), which is often indicated to be “very” or “extremely” demanding ( Shauman *et al.,* 2018), taking time away from conducting research and publishing scholarly work.

It is critical to reduce the pressure and stress faculty feel from their job demands as it is a barrier to their engagement in health and wellness activities. Possible contributors could be the innumerable changes in clinical medicine in recent years, such as electronic medical records and accompanying clerical burdens being easily accessed from home, blurring the boundary between work and home responsibilities ( Shanafelt *et al.,* 2016). The COVID-19 crisis has further blurred the boundaries of work and home, now with the additional task of helping children with virtual learning. Asking women to take on the responsibilities alone of reducing the amount of strain-based work-to-family conflict they experience, is unrealistic. Individuals at higher levels of departmental/division/organization leadership will need to be instrumental in reducing such demands.

### Part-Time

It will be important to examine the reasons faculty choose to work part-time. Women may do so earlier in their career as an attempt to reduce stress in their work lives ( Linzer and Harwood, 2018), possibly due to family demands. The finding of lower work-to-family conflict in women faculty who are part-time, compared to full-time, despite working an average of 41.9 hours is an interesting one to examine. A reduction in hours could help, although would not completely address the strain caused by work demands. Part-time women faculty, despite working an average of 41.9 hours a week have a lower work-to-family conflict than full-time faculty. It may be that working part-time reduces the amount of non-essential job tasks (e.g. committee work) which contribute to strain-based work-to-family conflict or that being part-time allows some personal control over hours at work. It should be noted that none of the women faculty who are single were part-time and the choice of part-time may only be an option for a two income family.

Lower work-to-family conflict may be accompanied by longer times to promotion. Part-time faculty may be required to focus primarily on clinical work, instead of research, either voluntarily or due to departmental practices. The consequence of reduced academic time and scholarly activity will lead to a longer length of time to promotion. It can be challenging to negotiate to protect academic time when reducing hours, as departments rely on clinical revenue for financial stability.

The implications for changes in departmental culture should be aimed at reducing the impact on work-to-family conflict, including structured mentoring programs. These could include innovative mentoring approaches, such as creating sessions using vertical and peer mentoring which focus on topics specific to women in medicine. Such sessions have been shown to: provide mentors and role models, offer a supportive environment, provide discussions pertinent to both personal and professional development, and expand networking opportunities to junior faculty ( Welch *et al.,* 2012). Also important will be to identify the promotional barriers associated with the decision to work part-time. Overall, future studies should consider diversity factors (e.g. race, religion, age), in addition to sex, as these additional barriers may further inhibit women from achieving promotion. While the departmental/division culture was important, it was not sufficient to completely mitigate work-to-family conflict. Continued efforts for practice reform in reducing inefficient workflow is crucial in reducing work-to-family conflict and reducing barriers to academic career success for all faculty.

### Limitations

There are limitations to this study. The study was completed at one institution, limiting the generalizability of the results. Additionally, we had an overall survey response rate of 48% (n=88 of 183) of potential female faculty respondents, with a much higher response rate from one department (FM=80.6%). The responses of those who chose to participate may not be representative of all who were eligible to respond.

## Conclusion

Existing strategies to improve the work climate should be enhanced, based on the survey findings comparing the perceptions and experiences of faculty with the highest work-to-family conflict compared with the lowest, as well as the impact of the strain of work demands on women’s engagement with their family and their own health. These include mentoring/coaching, guidance on research /publication opportunities, addressing prescriptive gender norms, and positive counter-stereotype imaging ( Carr *et al.,* 2019). The following should also be considered, decreasing women’s isolation from colleagues, promoting leadership and recognition, and identifying women leaders in the department for committees ( Fried *et al.,* 1996). Flexible use of hours, including use of their own sick time for family leave, and encouraging leadership to factor in childcare and other responsibilities in the timing of mandatory meetings, may also reduce women’s perceived need to work part-time. Women academic faculty may gain increased benefits from women mentors, as they can serve as role models for their junior counterparts in areas such as workplace communication, boundary setting, negotiation, and work-life balance ( DeCastro *et al.,* 2013). It is in an employer’s best interest to have a healthy workforce with low levels of burnout. Moreover, education could be provided to male colleagues holding leadership positions, on the unique aspects of work-to-family conflict women faculty members face. This can foster collaborative relationships in creating innovative solutions and prepare them to be better equipped as mentors to women faculty.

## Take Home Messages

• High work role overload and longer work hours are associated with women faculty feeling too stressed to fully engage with and enjoy time with their family or focus on their own health and wellness.
• Those with high work-to-family conflict report not having equal access to opportunities, support for work-life balance, or freedom from gender bias.

## Notes On Contributors

Julie A. Radico, PsyD ABPP is an Assistant Professor in the Department of Family Medicine and a clinical psychologist at the Penn State College of Medicine and Penn State Health, in Hershey, PA United States of America.

Tamara K. Oser, MD is Associate Professor in the Department of Family Medicine at the University of Colorado School of Medicine Anschutz, Clinical Professor in the Department of Family and Community Medicine at Penn State College of Medicine, and Director of the High Plains Research Network, United States of America. ORCiD: https://orcid.org/0000-0002-0405-3420

Tracy B. Fausnight, MD, FAAAAI, FACAAI is Professor of Pediatrics and Medicine, Medical Director of Pediatric Specialties, and Division Chief of Allergy and Immunology in the Department of Pediatrics at the Penn State College of Medicine and Penn State Health, in Hershey, PA United States of America.

Arthur Berg, PhD is a faculty biostatistician and his recent methodological research is in the area of Bayesian statistics at the Penn State College of Medicine and Penn State Health, in Hershey, PA United States of America. ORCiD: https://orcid.org/0000-0002-4097-7348

Ann Ouyang, MD is Professor of Medicine and Vice Chair for Faculty Promotion and Mentoring in the Department of Medicine at the Penn State College of Medicine and Penn State Health, Hershey, PA United States of America.

Shou Ling Leong, MD, is Assistant Dean for Pathways Innovation, Director of 3+ Accelerated Pathway Program, Associate Vice Chair for Education, Department of Family and Community Medicine at the Penn State College of Medicine and Penn State Health, in Hershey, PA United States of America.

## Appendices

Appendix 1: Sample survey recruitment language

Dear Women Faculty,

As you may be aware, Dr. Ruffin has convened a sub-committee specifically to explore women’s experience with achieving promotion in the Department of Family & Community Medicine in an effort to identify associated barriers, motivators, and the realities facing women in our department. We are conducting a preliminary survey to address concerns related to women’s promotion with respect to equal access to resources and opportunities, support for work-life balance, management of bias, department chair support, etc. We hope you will help by voluntarily completing a 20 minute anonymous survey. Any possibly identifying information will be removed before compiled data is shared.The survey can be found here (see Supplementary File 1).

Thank you for your help and support. Please contact us with any questions.
