# Supplementary material for: Factors that Influence Work Family Conflict for Women Faculty
Source: MedEdPublish (2016). 2021 Mar 8;10:63. [Version 1] doi: 10.15694/mep.2021.000063.1 (PMC10939630; doi:10.15694/mep.2021.000063.1)
Supplement: Peer review history

# Reviewer report: Reviewer response for version 1

**DOI:** 10.21956/mep.18964.r27152

**Published:** 2021-03-18

**Authors:** Keith Wilson

**Affiliations:** 1 Dalhousie University

This review has been migrated. The reviewer awarded 4 stars out of 5 The authors of this timely research project set out to explore the roles of work overload, conflicts between work and family and culture on academic success of women. The study involved a survey of women assistant and associate professors eligible for promotion in three departments at Penn State College of Medicine. Although the response rate was 48%, the results are absolutely worth exploring.One of the most important aspects of this paper is how it highlights concerning system problems that women experience in their efforts to be promoted academically. The authors explore challenges faced including discomfort some women with high work-to-family conflict have feeling supported in their workplace. Similarly, these women feel that family demands are not taken into account in scheduling and that it is expected that work should be a main focus. This study involves a single institution so generalizability could be hampered – I suspect that next steps for the researchers would be to compare across jurisdictions and possibly country boundaries. From the description in the paper, the survey included subscales from previously validated measures of conflict between work and family. It is not clear if the full instrument was used and if not, revalidation in this context may have been helpful. Similarly, three questions were used from Peterson’s role overload scale. For sake of completeness, it would be helpful to include which statistical correlation coefficient was used for the calculations. Similarly, the reader may want to know the t- and chi-square values that were associated with the results in the text.The possible explanations for the results discussed by the authors seem plausible and would benefit from further exploration in future studies. Radico et al should be commended for such an interesting and insightful paper – it should be widely read by faculty and policy makers alike to ensure the problems presented are addressed appropriately!

Reviewer Expertise:

NA

No decision status is available

# Reviewer report: Reviewer response for version 1

**DOI:** 10.21956/mep.18964.r27151

**Published:** 2021-03-09

**Authors:** Julie Hunt

**Affiliations:** 1 Lincoln Memorial University

This review has been migrated. The reviewer awarded 4 stars out of 5 This paper discusses a survey of women faculty at the ranks of Assistant and Associate Professor in primary care practice at a single medical school in the U.S. and how their working hours and culture impact their work-family conflict. The authors have done a good job of pointing out why this topic is worth researching: women are promoted at reduced rates as compared with their male counterparts. The survey instrument is validated, and the response rate is acceptable (48%), although disproportionate among the departments they distributed it to. The results are worthy of discussion. Some notable findings include the relatively high number of hours worked by even the part-time doctors (>40 hours/week), and the even higher number of hours worked by full-time doctors (approaching 60 hours/week)--no wonder the women have difficulty with work-family conflict. I would like to have seen some discussion of how these figures compare to other reported weekly work hours reported in the literature, and discussion of whether it is these numbers more than work culture that needs to change. The addition of discussion of demands placed on women outside the workplace may also have been helpful - e.g. women still do the bulk of the caregiving duties at home, as well as the work of keeping a household, despite living in a 2-income home. Until there is a widespread change in these factors - fewer working hours or a more egalitarian division of labor at home - women are likely to continue to experience work-family conflict at a level that exceeds that of men, and that makes this paper relevant across most of modern society.

Reviewer Expertise:

NA

No decision status is available
